# Plasma human growth cytokines in children with vasovagal syncope

**DOI:** 10.3389/fcvm.2022.1030618

**Published:** 2022-10-14

**Authors:** Yuanyuan Wang, Yaru Wang, Bing He, Chunyan Tao, Zhenhui Han, Ping Liu, Yuli Wang, Chaoshu Tang, Xueqin Liu, Junbao Du, Hongfang Jin

**Affiliations:** ^1^Department of Pediatrics, Peking University First Hospital, Beijing, China; ^2^Department of Pediatrics, People’s Hospital of Wuhan University, Hubei, China; ^3^Department of Cardiology, Children’s Hospital of Kaifeng, Kaifeng, China; ^4^Key Laboratory of Molecular Cardiovascular Sciences, Ministry of Education, Beijing, China; ^5^Department of Physiology and Pathophysiology, Health Science Centre, Peking University, Beijing, China

**Keywords:** vasovagal syncope, human growth cytokine, plasma sample, children, validation

## Abstract

**Purpose:**

The study was designed to investigate the profile of plasma human growth cytokines in pediatric vasovagal syncope (VVS).

**Materials and methods:**

In the discovery set of the study, plasma human growth cytokines were measured using a Quantiboby Human Growth Factor Array in 24 VVS children and 12 healthy controls. Scatter and principal component analysis (PCA) diagrams were used to describe the samples, an unsupervised hierarchical clustering analysis was used to categorize the samples. Subsequently, the cytokines obtained from the screening assays were verified with a suspension cytokine array in the validation set of the study including 53 VVS children and 24 controls. Finally, the factors associated with pediatric VVS and the predictive value for the diagnosis of VVS were determined.

**Results:**

In the discovery study, the differential protein screening revealed that the plasma hepatocyte growth factor (HGF), transforming growth factor b1 (TGF-b1), insulin-like growth factor binding protein (IGFBP)-4, and IGFBP-1 in children suffering from VVS were higher than those of the controls (all adjust *P*- value < 0.05). However, the plasma IGFBP-6, epidermal growth factor (EGF), and IGFBP-3 in pediatric VVS were lower than those of the controls (all adjust *P*- value < 0.01). Meanwhile, the changes of 7 differential proteins were analyzed by volcano plot. Unsupervised hierarchical cluster analysis demonstrated that patients in the VVS group could be successfully distinguished from controls based on the plasma level of seven differential proteins. Further validation experiments showed that VVS patients had significantly higher plasma concentrations of HGF, IGFBP-1, and IGFBP-6, but lower plasma concentrations of EGF and IGFBP-3 than controls. The logistics regression model showed that increased plasma concentration of HGF and IGFBP-1 and decreased plasma concentration of EGF were correlated with the development of pediatric VVS. ROC curve analysis showed that the abovementioned 3 proteins were useful for assisting the diagnosis of VVS.

**Conclusion:**

Plasma human growth cytokine profiling changed in pediatric VVS. Elevated plasma concentrations of HGF and IGFBP-1, and decreased EGF were associated factors in the development of pediatric VVS. The abovementioned three proteins are helpful for the diagnosis of pediatric VVS.

## Introduction

Syncope is common in children and is thought to be a transient loss of consciousness due to transient cerebral hypoperfusion with the loss of muscle tone and a failure to maintain posture. Vasovagal syncope (VVS) accounts for 60–70% of pediatric syncope (1–3). Syncopal recurrent episodes can severely affect physical and psychological health and even result in unpredictable injuries to children due to falls (4).

A previous study demonstrated that the flow-mediated vasodilation was enhanced in children with VVS at the supine position (5), suggesting that children and adolescents with VVS had excessive vasodilation (6, 7). Similarly, levels of hydrogen sulfide in plasma and erythrocytes were also remarkably increased in supine VVS children (8, 9). Furthermore, serum endothelin 1 (ET-1) was significantly increased in pediatric VVS at supine and initial tilt positions (10), whereas another study suggested that plasma ET-1 was not increased during tilt and before syncope (11). The imbalance between vasoconstriction and vasodilation may be associated with neurohumoral dysregulation, but the regulatory mechanisms for vascular dysfunction in children with VVS remain unclear. It is reported that vascular growth cytokines play an essential role in regulating vasomotor function directly or indirectly. For instance, epidermal growth factor (EGF) (12), platelet-derived growth factor (PDGF) (13), and fibroblast growth factor (FGF) (14) were associated with vasoconstriction. On the contrary, insulin-like growth factor binding protein (IGFBP)-1 (15) and hepatocyte growth factor (HGF) (16) were related to vasodilation.

However, the expression profile of growth cytokines in the plasma and the possible pathophysiologic and clinical significance in the development of pediatric VVS remain unclear. In recent years, cytokine antibody arrays enable us to precisely identify the expression of multiple cytokines simultaneously. At present, it has been extensively applied in the research field because of its advantages of high detection sensitivity and specificity, and the high throughput of arrays (17, 18). The suspension cytokine array can also be used to verify the selected biomarkers with very low protein concentrations by low-dose plasma samples. Therefore, in this study, the cytokine antibody arrays were used to determine the expression profile of cytokines, and the suspension cytokine arrays were used to validate the changes in growth cytokine expression to clarify whether plasma growth cytokines were involved in the development of pediatric VVS and its possible clinical implication.

## Materials and methods

### Study participants

**FIGURE 1:**
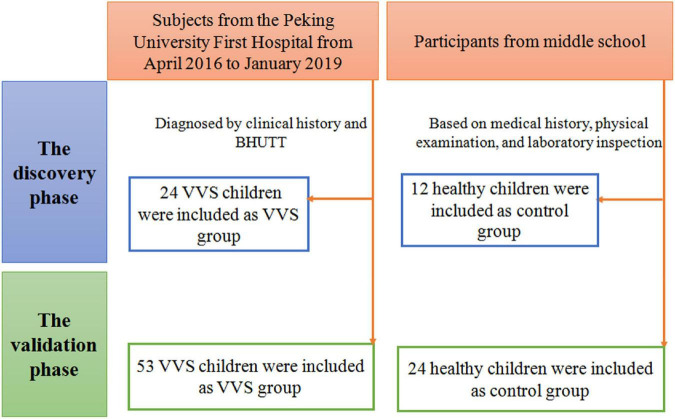
Flow-chart of patient inclusion. In the discovery phase, a total of 36 subjects were included, including 24 cases in the VVS group and 12 cases in the control group. In the validation phase, a total of 77 cases were included, including 53 cases in the VVS group and 24 cases in the control group. BHUTT, baseline head-up tilt test; VVS, vasovagal syncope.

As shown in Figure 1, the VVS group consisted of pediatric patients with VVS admitted to the Peking University First Hospital from April 2016 to January 2019 and the control group included healthy children identified as healthy based on their medical history, physical examination, and laboratory inspection from a middle school. In the discovery set of the study, there were 24 children in the VVS group and another 12 children in the control group. While, in the validation set of the study, 53 children were included in the VVS group and another 24 children served as controls.

Inclusion criteria of the study patients: (1) children aged 5–18 years; (2) patients diagnosed with VVS; (3) with adequate plasma samples. The following patients were excluded: (1) with incomplete clinical data; (2) hemolysis of plasma specimens; (3) patients with neurological, metabolic, cardiovascular, or mental disease.

The diagnostic criteria of pediatric VVS are as follows (19, 20): (1) with predisposing factors such as persistent standing, or a sultry environment; (2) with syncope or presyncope clinically; (3) with a positive response appearance in baseline head-up tilt test (BHUTT): significant blood pressure (BP) drop [systolic BP (SBP) ≤ 80 mmHg or diastolic BP (DBP) ≤ 50 mmHg or mean BP drop by ≥ 25%]; and (or) heart rate (HR) decline (HR < 75 bpm for those at 4–6 years old, HR < 65 bpm for those at ∼8 years old or HR < 60 bpm for those over 8 years old; and (4) excluding other possible causes of syncope, such as cardiac syncope and cerebrovascular diseases. The children in the control group met the following conditions: (1) no syncope or presyncope occurred clinically; and (2) no positive findings on physical and laboratory examinations. The research was permitted by the Biomedical Research Ethics Committee of Peking University First Hospital (2018 [112]) and followed the Declaration of Helsinki. The informed consent was approved by the Committee of Peking University First Hospital and was acquired from the subjects’ guardians.

### BHUTT

Subjects fasted for more than 4 h and were asked to avoid taking any drugs that affected autonomic nervous function before the test. Firstly, the patient lay on his back on a tilt bed (SHUT-100A, Standard, Jiangsu and ST-711, Juchi, Beijing, China) for at least 10 min. The multi-lead electrocardiogram (ECG) monitor (General Electric, New York, NY, USA) was used to continuously measure HR and ECG, and a Finapres Medical System-FMS (FinometerPRO, FMS Company, Netherlands) was applied to dynamically monitor BP in a warm and quiet condition. Subsequently, the tilt bed was tilted at 60^°^, and the ECG, HR, and BP were measured until the appearance of a positive response, or otherwise, the child finished the whole test duration of 45 min if no positive response was observed (21, 22).

### Plasma specimen collection

Blood samples of the participants were collected before the patients with BHUTT. Totally 2 ml of venous blood was obtained from subjects in the VVS and control groups under fasting, quiet and supine states. The blood was anticoagulated with ethylene diamine tetraacetic acid and centrifuged at 4°C at 2,000 r/min for 20 min. Subsequently, the supernatant was acquired and added with aprotinin in a ratio of 1:100. The plasma was then immediately frozen at −80°C until further detection.

### Cytokine antibody arrays

Plasma levels of human growth cytokines in 24 VVS patients and 12 healthy children were detected by cytokine antibody arrays. The Human Growth Factor Array 1 (QAH-GF-1, Quantiboby; RayBiotech; Norcross, GA, USA) was used to detect 40 selected cytokines simultaneously. The assay was carried out in strict accordance with the instructions and sandwich immunoassay principle. Eighty microliters (μl) of detection antibody cocktail were applied to each well to immobilize the antibody targeting the selected cytokine at a specific location on the surface of the array glass. Cytokines present in the plasma were recognized by the corresponding antibodies and conjugated with Cy3 equivalent dye-conjugated streptavidin to detect the binding cytokines. The signals can be visualized using fluorescent dye by InnoScan 300 Microarray Scanner (Innopsys, Carbonne, France), and were detected with a Cy3 (532 nm) wavelength (green channel). The catalog number of the Q-Analyzer Tool specific for this array is QAH-GF-1-SW.

### Suspension cytokine arrays

The concentrations of cytokines identified in the cytokine antibody array were further validated in plasma samples from 53 VVS patients and 24 control children using the Human Premixed Multi-Analyte Kit (Luminex 200 system; Cat: LXSAHM-01 [L130457] and LXSAHM-07 [L130460]; Luminex Corporation, Austin, TX, USA) according to the manual. The selected cytokines included IGFBP-6, EGF, HGF, IGFBP-1, IGFBP-4, and IGFBP-3. An amount of 50 μl sample was added to each well and incubated with RD2-1 diluted magnetic beads for 2 h in the dark. After three washes using a Magnetic Plate Washer (Tecan), the samples were incubated with the diluted biotin-antibody cocktail for 1 h in a dark environment. After discarding the biotin-antibody cocktail, it was rewashed with a Magnetic Plate Washer (Tecan) three times. And the streptavidin-conjugated phycoerythrin was used to visualize the captured cytokines and the specific concentration of each cytokine was calculated automatically using a Luminex 200 array reader (Luminex Corporation, Austin, TX, USA).

### Statistical analysis

As the analysis software, language R (version number 4.1.2) was used in the screening of the differentially expressed proteins. The analysis method is moderated *t*-statistics, and the data package is limma. Adjust *P*- value (Adj. *P*. Val, BH method corrected *P*- value) < 0.05 and fold change > 1.2 or < 0.83 [absolute logFC (expression difference multiple in 2 is the bottom) > 0.263] were used to select the differential proteins. After the screening of the differential proteins, it was visualized by volcano plot, with the drawing function being ggplot2, and the data packet ggfortify. The principal component analysis (PCA) plot adopts the prcomp function, the drawing function is autoplot, and the data packet is ggfortify. The cluster heatmap analysis method is the heatmap.2 function and the data packet is gplots.

In the analysis of the clinical characteristics of the study population and biomarkers verification, the software of SPSS 23.0 (IBM, Armonk, NY, USA) was carried out. Data normality was verified by the Kolmogorov-Smirnov test. The measurement variables with a normal distribution were displayed with mean ± standard deviation (SD). Otherwise, the data were displayed with median (P_25_, P_75_), respectively. When the two sets of measurement data met the normal distribution, the independent sample *t*- test was applied to compare the difference between the groups, or otherwise, the Mann-Whitney U test was adopted. The categorical variables were displayed with the number of cases. Categorical variables were compared with the χ^2^ test between groups. The multivariate analysis was achieved by a logistic regression model (conditional forward method). *P*- values of <0.05 were thought to be remarkably different.

## Results

### Participants information

In the discovery set of the study, there were 24 patients in the VVS group and 12 healthy children in the control group, and the mean age was 11.2 ± 2.5 and 10.5 ± 0.8 years, respectively. The validation study was conducted among 53 children in the VVS group and 24 children in the control group, and their median age was 10.0 (8.0, 13.0) years and 10.0 (10.0, 11.0) years, respectively. There was no statistical significance in gender, age, height, weight, supine HR, SBP, and DBP between the two groups in the discovery set of the study or the validation set of the study (Tables 1, 2).

**TABLE 1:** Clinical characteristics of the study population in the discovery study.

Groups	VVS (*n* = 24)	Control (*n* = 12)	χ^2^/*t*	*P*-value
Sex (M/F) (*n*)	10/14	7/5	0.348	0.555
Age (years old)	11.2 ± 2.5	10.5 ± 0.8	1.250	0.221
Height (cm)	153.1 ± 16.2	149.3 ± 3.2	1.105	0.279
Weight (kg)	44.2 ± 13.1	43.5 ± 5.6	0.210	0.835
Supine HR (bpm)	83.8 ± 17.3	82.1 ± 8.1	0.315	0.755
Supine SBP (mmHg)	110.0 ± 11.0	112.0 ± 7.0	−0.449	0.657
Supine DBP (mmHg)	67.0 ± 7.0	63.0 ± 9.0	1.706	0.097

VVS, vasovagal syncope; HR, heart rate; SBP, systolic blood pressure; DBP, diastolic blood pressure.

Normally distributed data are displayed with mean ± standard deviation; Categorical variables are presented by numbers.

**TABLE 2:** Clinical characteristics of the study population in the validation study.

Groups	VVS (*n* = 53)	Control (*n* = 24)	χ^2^/*Z*/*t*	*P*-value
Sex (M/F) (*n*)	21/32	14/10	2.333	0.127
Age (years old)	10.0 (8.0, 13.0)	10.0 (10.0, 11.0)	−0.190	0.849
Height (cm)	152.0 (132.5, 161.0)	146.0 (139.3, 150.8)	−0.991	0.322
Weight (kg)	40.6 ± 12.5	39.4 ± 9.2	0.438	0.663
Supine HR (bpm)	81.8 ± 14.2	82.5 ± 8.4	−0.228	0.820
Supine SBP (mmHg)	107.0 (100.5, 116.5)	110.5 (106.0, 115.0)	−0.886	0.376
Supine DBP (mmHg)	66.4 ± 7.6	64.8 ± 9.1	0.806	0.423

VVS, vasovagal syncope; HR, heart rate; SBP, systolic blood pressure; DBP, diastolic blood pressure.

Normally distributed data are displayed with mean ± standard deviation and non-normally distributed data are presented by median (P_25_, P_75_); Categorical variables are displayed with numbers.

### Discovery of plasma protein profile between two groups

**FIGURE 2:**
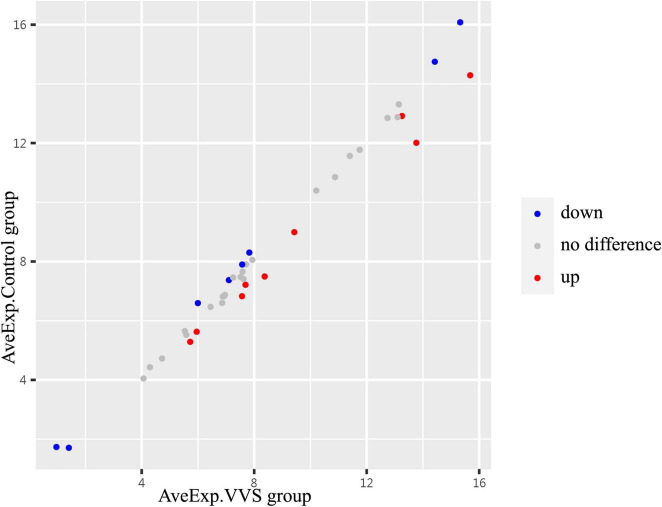
The centralized tendency of two sets of chip data was evaluated by scatter plot. Red presents up-regulation, blue presents down-regulation, and gray presents no difference. The X- and Y-axis show the average plasma protein concentrations of VVS group and of the control group, respectively. AveExp.VVS group, the average plasma protein concentration of the VVS group; AveExp.Control group, the average plasma protein concentration of the control group.

**FIGURE 3:**
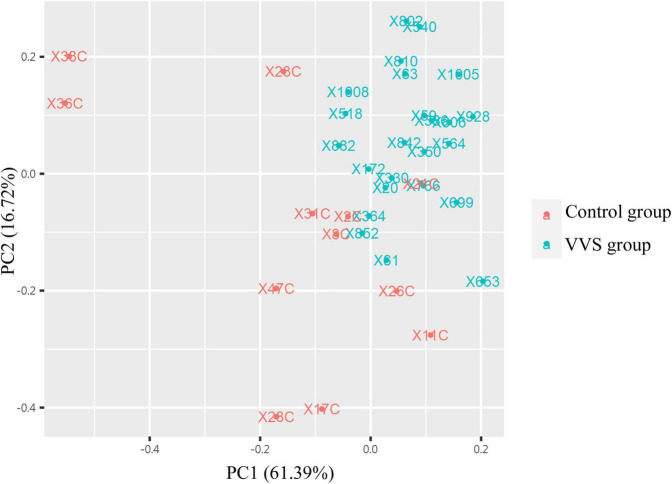
The analysis of principal component analysis (PCA) between the two groups. PCA analysis showed that the data tended to clustered distribution and were distributed in two-dimensional space, suggesting that samples have good representativeness and biological repeatability, and are suitable for further analysis. The red color presents the control group, and the green color presents the VVS group. VVS, vasovagal syncope.

**FIGURE 4:**
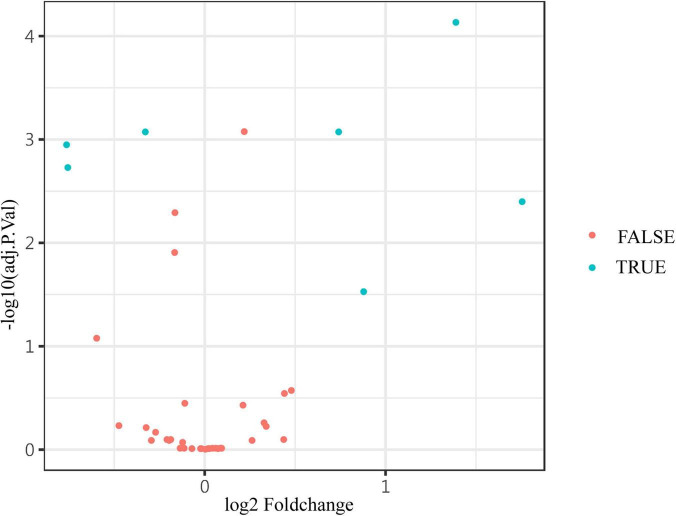
The volcano plot showed the expression of differential proteins. The differential levels of proteins (blue) and non-differential substances (red) were identified by Adj. *P*. values < 0.05, and fold change > 1.2 or <0.83 (absolute logFC > 0.263) in the volcano plot. The Adj. *P*. values were converted to -log10 (Adj. *P*. value) so that the higher the values on the y-axis, the more remarkably the difference in protein level changes with smaller Adj. *P*. values. The fold change is log-converted, so negative values and positive values indicated a decrease and an increase in protein levels, respectively. Blue (true) and red (false) represent significant and non-significant expression proteins, respectively.

**FIGURE 5:**
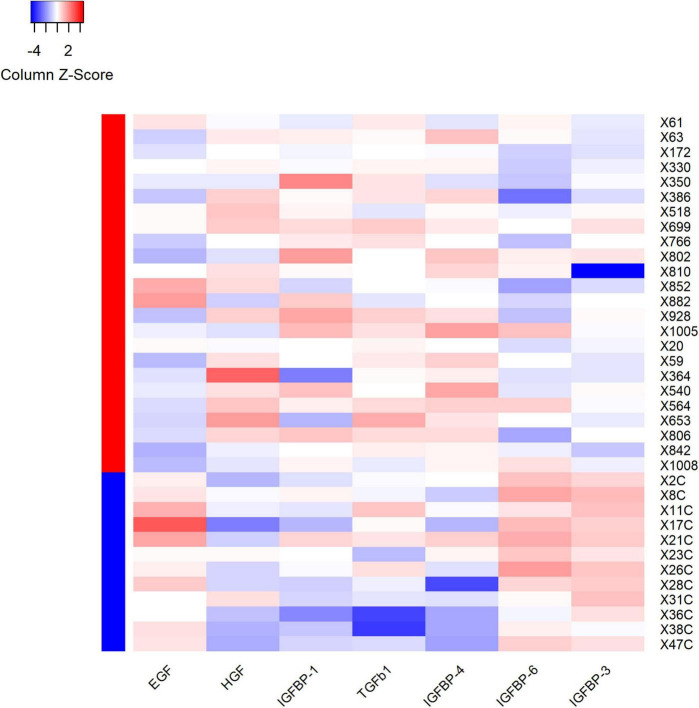
Heatmap showed the outcomes of hierarchical cluster analysis (HCA) independently performed on both samples’ and variables’ dimensions, for the seven differential expression proteins. Each row in the figure represents a subject, and each column represents a protein. The average protein expression level of the same patient was used as the benchmark. If the expression level of protein is higher than the average level, it is considered positive and marked as red; on the contrary, the expression level of protein is considered negative and marked as blue. The red and blue colors in the left column stand for the VVS group and control group, respectively. For plasma HGF, IGFBP-1, TGF-b1 and IGFBP-4, the proportion of red color in the VVS group was significantly higher than that in the control group, which means that the above four proteins in the VVS group were significantly higher than those in the control group. However, the proportion of blue color of plasma EGF, IGFBP-3, and IGFBP-6 proteins in the control group was remarkably higher than that in the VVS group, which means that the above three plasma protein concentrations were remarkably higher in the control group than those in the VVS group. IGFBP, insulin-like growth factor binding protein; HGF, hepatocyte growth factor; EGF, epidermal growth factor; TGF-b1, transforming growth factor b1.

**FIGURE 6:**
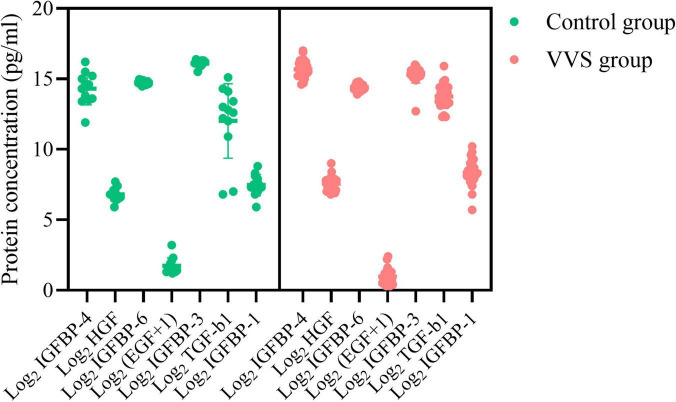
Plasma concentration profiles of seven differential proteins in the control group and the VVS group. The longitudinal axis represents the seven proteins, and the transversal axis represents the protein concentration. The EGF is expressed on a log2-scale by adding 1 to the original plasma concentration; and other biomarker plasma concentrations of the assessed proteins are expressed on a log2-scale. The green color and red color of the graph stand for the subjects in the control group and the VVS group, respectively. VVS, vasovagal syncope; IGFBP, insulin-like growth factor binding protein; HGF, hepatocyte growth factor; EGF, epidermal growth factor; TGF-b1, transforming growth factor b1.

In the discovery test, a cytokine antibody array was used for comparing the profile of human growth factors between the two groups. To show the distribution of test values after the normalized quantile and guarantee the accuracy and reliability of data, the scatter plot (Figure 2) and PCA plot (Figure 3) were employed to assess the distribution of the data, and this evaluation showed relatively good representativeness of the applied samples in the study. Seven differential proteins were identified between the VVS group and control group by volcano plot analysis (differentially altered proteins were identified as those with Adj. *P*. Val. < 0.05, and fold change > 1.2 or <0.83 (absolute logFC > 0.263) (Table 3 and Figure 4). Specifically, the VVS patients had higher plasma protein concentrations of IGFBP-4, transforming growth factor b1 (TGF-b1), HGF, and IGFBP-1, but lower levels of IGFBP-6, EGF, and IGFBP-3 than the controls. Unsupervised hierarchical cluster analysis demonstrated that children with VVS can be distinguished from healthy children based on the plasma concentration of seven differential proteins (Figure 5). The plasma concentration profile of the seven different proteins between the control group and the VVS group was shown in Figure 6. The compared results of the other 33 proteins between the two groups were detailed in (Supplementary Table 1).

**TABLE 3:** Plasma protein profile between VVS and control groups in the discovery study.

Biomarkers	VVS group	Control group	Log FC	*P*-value	Adj. *P*. Val	Log_2_ (FC)
Log_2_ IGFBP-4 (pg/ml)	15.7 ± 0.6	14.3 ± 1.1	1.389	<0.001	<0.001	2.619
Log_2_ HGF (pg/ml)	7.6 ± 0.5	6.8 ± 0.5	0.741	<0.001	<0.001	1.671
Log_2_ IGFBP-6 (pg/ml)	14.4 ± 0.2	14.8 ± 0.1	−0.328	<0.001	<0.001	0.797
^&^Log_2_ (EGF + 1) (pg/ml)	0.9 (0.5, 1.3)	1.6 (1.3, 2.1)	−0.764	<0.001	0.001	0.589
Log_2_ IGFBP-3 (pg/ml)	15.4 (15.2, 15.6)	16.2 (16.0, 16.3)	−0.757	<0.001	0.002	0.592
Log_2_ TGF-b1 (pg/ml)	13.7 (13.3, 14.4)	12.7 (11.2, 13.9)	1.755	<0.001	0.004	3.376
Log_2_ IGFBP-1 (pg/ml)	8.4 ± 1.0	7.5 ± 0.8	0.879	0.007	0.030	1.839

IGFBP, insulin-like growth factor binding protein; HGF, hepatocyte growth factor; EGF, epidermal growth factor; TGF-b1, transforming growth factor beta-1; Adj. *P*. Val, BH method adjusted *p*-value; Normally distributed data are displayed with mean ± standard deviation and non-normally distributed data are presented by median (P_25_, P_75_); Log_2_(FC), Log_2_ (fold change).

^&^To prevent the calculation site from being negative, the EGF is expressed on a log2-scale by adding 1 to the original plasma concentration.

And other biomarkers plasma concentrations of the assessed proteins are expressed on a log2-scale.

Differentially expressed proteins (DEPs) are defined as those with Adj. *P*. Val < 0.05, and fold change > 1.2 or <0.83 (absolute logFC > 0.263).

### Validation of plasma protein profile between two groups

To validate the data screened by cytokine antibody array, the human suspension cytokine array was used for measuring the levels of growth cytokines. As shown in Table 4, the data of plasma protein concentrations or fluorescence suggested that plasma levels of HGF, IGFBP-6, and IGFBP-1 were higher in the VVS patients than in the controls. However, the result of plasma EGF and IGFBP-3 were lower in the VVS patients than in the controls. The following cytokines, IGFBP-4, and TGF-b1 could not be detected because the concentrations were too low or below the detection range of the standard curve.

**TABLE 4:** Plasma proteins validation in VVS and control groups.

Biomarkers	VVS group (*n*)	Control group (*n*)	*t*/*Z*	*P*-value
Log_2_ HGF (pg/ml)	6.5 ± 0.5	5.8 ± 0.6	5.001	<0.001
Log_2_ IGFBP-6 (pg/ml)	16.8 (16.6, 17.0)	16.6 (16.3, 16.8)	−2.364	0.018
^&^Log_2_ (EGF + 1) (pg/ml)	2.5 (1.7, 3.2)	4.4 (3.9, 5.0)	−5.318	<0.001
Log_2_ IGFBP-1 (pg/ml)	13.6 ± 1.1	13.0 ± 0.7	2.649	0.010
^#^Log_2_ IGFBP-3 (pg/ml)	11.6 ± 0.5	11.8 ± 0.5	−2.013	0.048

^#^The biomarkers expressed by fluorescence signal-BKG.

^&^The EGF is expressed on a log2-scale by adding 1 to the original plasma concentration; and other biomarker plasma concentrations of the assessed proteins are expressed on a log2-scale.

Normally distributed data are displayed with mean ± standard deviation and non-normally distributed data are presented by median (P_25_, P_75_).

HGF, hepatocyte growth factor; IGFBP, insulin-like growth factor binding protein; EGF, epidermal growth factor.

### Plasma growth cytokines were associated factors for the development of pediatric vasovagal syncope

Demographic characteristics and the cytokines with a *P*- value < 0.1 in univariate analysis were introduced into multivariate logistic analysis, including gender, age, BMI, HGF, EGF, IGFBP-6, IGFBP-1, and IGFBP-3. According to the results of multivariate logistics regression analysis, increased HGF and IGFBP-1 and decreased EGF were associated factors in the development of pediatric VVS (Table 5).

**TABLE 5:** Logistic multivariate regression analysis of variables.

Characteristics	B	SE	Wald	*P*-value	OR (95% CI)
Log_2_ (EGF + 1)	−0.995	0.332	8.994	0.003	0.370 (0.193–0.709)
Log_2_ HGF	2.051	0.701	8.572	0.003	7.778 (1.970–30.708)
Log_2_ IGFBP-1	1.227	0.528	5.395	0.020	3.412 (1.211–9.611)
Constant	−24.471	9.092	7.244	0.007	–

Characteristics enrolled in the logistic multivariate regression analysis: gender, age, BMI, log_2_ (EGF + 1), log_2_ IGFBP-6, log_2_ HGF, log_2_ IGFBP-1, and log_2_ IGFBP-3. HGF, hepatocyte growth factor; IGFBP, insulin-like growth factor binding protein; EGF, epidermal growth factor.

### Diagnostic value of plasma insulin-like growth factor binding protein-1, epidermal growth factor, and hepatocyte growth factor

**FIGURE 7:**
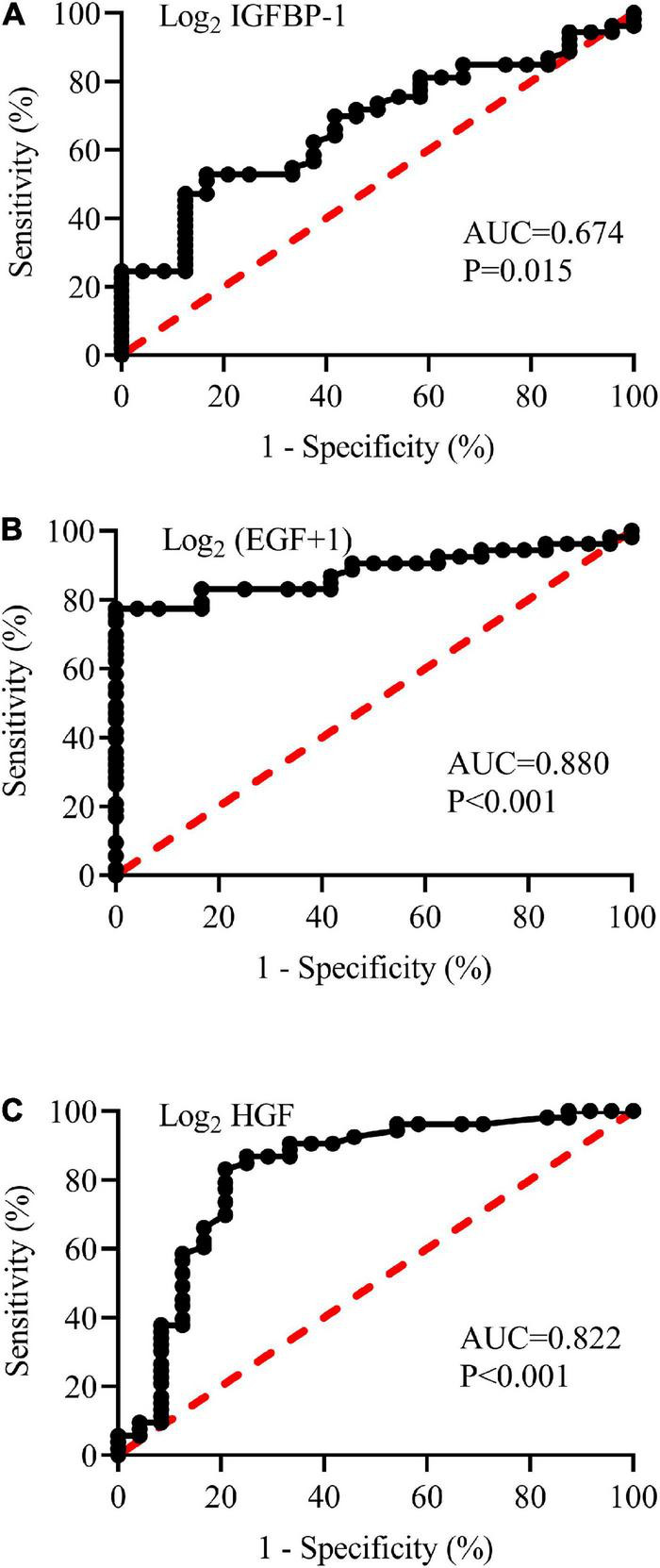
Receiver operating characteristic (ROC) curves of plasma concentrations of log_2_ IGFBP-1 **(A)**, log_2_ (EGF + 1) **(B)**, and log_2_ HGF **(C)** to analyze the prediction of the diagnosis of VVS in children. The longitudinal axis represents sensitivity to predict the diagnosis of pediatric VVS. The transversal axis represents the false positive rate (1-specificity) of the prediction. The 45° red dotted line is the reference line, indicating that the sensitivity is equal to the false positive rate, with no predictive value at all. The black curve is farther from the reference line and closer to the upper left corner of the graph, indicating higher diagnostic efficiency. The areas under the ROC curve of log_2_ IGFBP-1 **(A)**, log_2_ (EGF + 1) **(B)**, and log_2_ HGF **(C)** were 0.674, 0.880, and 0.822, respectively (all *P* < 0.05). The EGF is expressed on a log2-scale by adding 1 to the original plasma concentration; and other biomarker plasma concentrations of the assessed proteins are expressed on a log2-scale. AUC, area under the curve; IGFBP-1, insulin-like growth factor binding protein-1; HGF, hepatocyte growth factor; EGF, epidermal growth factor.

Based on the above results, the abilities of plasma IGFBP-1, EGF, and HGF to predict the diagnosis of pediatric VVS were further examined. As shown in Figure 7, the areas under the receiver operator characteristic curve of the logarithm of plasma IGFBP-1, EGF + 1, and HGF to base 2 were 0.674, 0.880, and 0.822, respectively. When the cut-off values of the logarithm of plasma IGFBP-1, EGF + 1, and HGF to base 2 were over 13.5 pg/ml, less than 3.4 pg/ml, or over 6.1 pg/ml, the sensitivities of predicting pediatric VVS were 52.8, 77.4, and 83.0%, respectively, and the specificities of predicting pediatric VVS were 83.3, 100, and 79.2%, respectively.

## Discussion

This study, for the first time, showed a characteristic plasma growth cytokine variation profile of a significantly increased plasma HGF, IGFBP-6, and IGFBP-1 and decreased plasma EGF and IGFBP-3. Plasma HGF, IGFBP-1, and EGF were associated factors for the development of pediatric VVS. The above findings would be extremely helpful in understanding the pathogenesis of VVS in children. In addition, plasma HGF, IGFBP-1, and EGF would be useful for assisting in the diagnosis of pediatric VVS.

Vasovagal syncope is a common entity of pediatric syncope and its recurrence can seriously affect patients’ physical and mental health. Therefore, it is crucial to elucidate the pathogenesis of pediatric VVS and further develop effective preventive strategies. However, till now, the pathogenesis of pediatric VVS has not been clear.

It is well known that excessive vasodilation is one of the pathogenesis of pediatric VVS (23), but the exact mechanism remains unclear. This study indicated that the plasma IGFBP-1 in pediatric patients with VVS was higher than that in controls, but how the IGFBP-1 was involved in pathogenesis in children with VVS remains unclear. Based on the previous studies, IGFBP-1 is a 30 kDa protein that can dilate blood vessels, reduce vascular resistance and increase cardiac blood flow (24, 25). It was reported that the production of basal NO was increased in transgenic IGFBP-1 overexpressed mice (26). IGFBP-1 can upregulate eNOS mRNA expression by stimulating the phosphorylation of endothelial nitric oxide synthase (eNOS) in the PI3K/AKT pathway. In the VVS cases, the high plasma concentration of IGFBP-1 would likely stimulate the upregulation of eNOS mRNA expression, which increased the release of NO from the vascular endothelium, resulting in excessive vasodilation (27, 28). However, whether and how IGFBP-1 is involved in the development of pediatric VVS merit further studies.

In addition to IGFBP-1, another cytokine, HGF, was also found to be significantly higher in the plasma of children with VVS than that of the controls. HGF is a multifunctional factor involved in the proliferation, differentiation, and regeneration of different cells *via* binding with its specific receptor c-met (29). In addition, HGF plays a part in the expression of eNOS in endothelial cells (30). This was also confirmed by western blot analysis in human saphenous vein endothelial cells (HGSVEC), suggesting that the upregulation of eNOS expression could be detected after 2 h by HGSVEC treated with HGF, reached its peak at 6–8 h, and remained at a high level 16 h later (31). In human coronary artery endothelial cells, the secretion of endothelin-1 was significantly reduced in a concentration-dependent manner in the presence of HGF (32). Similarly, HGF has been shown to induce rapid microvasodilation, which can be inhibited by NO blockers, suggesting that the short-term effect is the result of HGF-induced upregulation of eNOS expression (33). Therefore, we suspected that plasma HGF was likely involved in the pathogenesis of VVS and it might increase NO secretion by stimulating endothelial cells *via* eNOS phosphorylation, leading to excessive vasodilation and then facilitating the development of VVS. Whereas, further explorations are still needed to reveal the significance of disturbed growth cytokine HGF secretion in the pathogenesis of pediatric VVS.

Epidermal growth factor, a vasoconstrictor, was significantly decreased in the children with VVS in this study. EGF, a globular protein of approximately 6 kDa, composes of 53-amino acids (34) and promotes cell proliferation and differentiation to replace senescence and death cells with new cells (35). Also, it was found that EGF could induce the contraction of isolated rat pulmonary arteries in a dose-dependent manner; when low concentrations of EGF are insufficient to induce vasoconstriction, EGF can enhance the effect of angiotensin II and participate in the pathophysiological processes of pulmonary circulation (12). A study on chronic hypoxia (CH) disease showed that EGF receptors were necessary to enhance the depolarization-mediated vasoconstriction following CH, and EGF-induced pulmonary arteries contraction of CH by ROK-associated Ca^2+^ sensitization (36, 37). As a consequence, EGF is thought to play a part in vasoconstriction. In this study, the decreased plasma concentration of EGF in pediatric VVS suggested that the reduced vasoconstriction was likely to induce VVS under the circumstance of postural changes.

The data of multivariate logistic regression analysis showed that increased plasma concentrations of HGF and IGFBP-1 and decreased plasma concentrations of EGF were involved in the pathogenesis of pediatric VVS. In a word, the increased plasma HGF and IGFBP-1 with the vasodilating features might facilitate the syncopal episode, while increased plasma EGF might cause vasoconstriction in children and further reduce the possibility of VVS episodes to a certain degree.

However, there were still some limitations in this research, such as single-center and limited sample size. Moreover, we did not conduct studies on animal models to further investigate the possible mechanisms behind it. In the future, multicenter-based and large sample-sized studies should be performed to further elucidate the involvement of these cytokines in the pathogenesis of VVS. The exact role of cytokines discovered in the development of VVS needs to be illustrated *via* animal studies.

## Data availability statement

The original contributions presented in this study are included in the article/Supplementary material, further inquiries can be directed to the corresponding authors.

## Ethics statement

The studies involving human participants were reviewed and approved by Committee of Peking University First Hospital. Written informed consent to participate in this study was provided by the participants’ legal guardian/next of kin.

## Author contributions

YYW, CYT, HJ, XL, CST, and JD: conception and design of the study. YYW, BH, YRW, ZH, PL, XL, YLW, and CYT: collection of data. YYW, YRW, ZH, XL, HJ, and JD: data checking. YYW, BH, ZH, YRW, CST, HJ, and JD: analysis and interpretation of data. YYW, BH, and JD: drafting the manuscript. YRW, PL, BH, YLW, HJ, and JD: revising the manuscript. YYW, BH, YRW, HJ, and JD: final approval of the version to be submitted. All authors contributed to the article and approved the submitted version.
