# Supplementary material for: Plasma human growth cytokines in children with vasovagal syncope
Source: Front Cardiovasc Med. 2022 Oct 14;9:1030618. doi: 10.3389/fcvm.2022.1030618 (PMC9614254; doi:10.3389/fcvm.2022.1030618)
Supplement: Supplementary file 1 [file Table_1.doc]

Supplementary Material

**Appendix Table 1. Comparison of the other 33 plasma protein cytokines between VVS group and control group**

| Protein ID | AveExp.VVS | AveExp.Control | logFC | P value | Adj. P. Val | Fold change |
| --- | --- | --- | --- | --- | --- | --- |
| Log_2_ IGFBP-2 | 13.098 | 12.879 | 0.219 | <0.001 | 0.001 | 1.164 |
| Log_2_ MCSF R | 13.143 | 13.308 | -0.165 | 0.001 | 0.005 | 0.892 |
| Log_2_ SCF R | 11.400 | 11.566 | -0.166 | 0.003 | 0.012 | 0.891 |
| Log_2_ VEGF-D | 5.997 | 6.595 | -0.597 | 0.023 | 0.084 | 0.661 |
| Log_2_ BMP-4 | 7.692 | 7.213 | 0.479 | 0.080 | 0.268 | 1.394 |
| Log_2_ VEGF | 5.726 | 5.285 | 0.441 | 0.093 | 0.286 | 1.357 |
| Log_2_ EGF R | 12.741 | 12.851 | -0.110 | 0.125 | 0.356 | 0.927 |
| Log_2_ GDF-15 | 7.621 | 7.409 | 0.212 | 0.139 | 0.371 | 1.158 |
| Log_2_ bFGF | 5.954 | 5.626 | 0.328 | 0.220 | 0.550 | 1.255 |
| Log_2_ NT-3 | 7.827 | 8.301 | -0.474 | 0.249 | 0.586 | 0.720 |
| Log_2_ BMP-5 | 13.255 | 12.915 | 0.340 | 0.268 | 0.595 | 1.266 |
| Log_2_ Insulin | 7.574 | 7.897 | -0.324 | 0.291 | 0.612 | 0.799 |
| Log_2_ OPG | 7.100 | 7.372 | -0.272 | 0.339 | 0.679 | 0.828 |
| Log_2_ GH | 9.428 | 8.991 | 0.437 | 0.421 | 0.798 | 1.354 |
| Log_2_ SCF | 7.252 | 7.461 | -0.209 | 0.439 | 0.798 | 0.865 |
| Log_2_ PDGF-AA | 10.208 | 10.396 | -0.188 | 0.459 | 0.798 | 0.878 |
| Log_2_ IGF-1 | 7.709 | 7.906 | -0.196 | 0.495 | 0.814 | 0.873 |
| Log_2_ BDNF | 6.860 | 6.598 | 0.262 | 0.529 | 0.814 | 1.199 |
| Log_2_ TGFa | 1.411 | 1.706 | -0.295 | 0.529 | 0.814 | 0.815 |
| Log_2_ VEGF R3 | 7.933 | 8.055 | -0.122 | 0.574 | 0.850 | 0.919 |
| Log_2_ TGFb3 | 5.535 | 5.649 | -0.114 | 0.698 | 0.969 | 0.924 |
| Log_2_ PIGF | 4.292 | 4.428 | -0.136 | 0.711 | 0.969 | 0.910 |
| Log_2_ NGF R | 6.911 | 6.818 | 0.094 | 0.753 | 0.969 | 1.067 |
| Log_2_ AR | 6.960 | 6.874 | 0.086 | 0.775 | 0.969 | 1.061 |
| Log_2_ NT-4 | 7.518 | 7.475 | 0.044 | 0.795 | 0.969 | 1.031 |
| Log_2_ EG-VEGF | 5.585 | 5.523 | 0.062 | 0.799 | 0.969 | 1.044 |
| Log_2_ GDNF | 6.887 | 6.813 | 0.074 | 0.837 | 0.977 | 1.053 |
| Log_2_ FGF-7 | 7.588 | 7.658 | -0.071 | 0.878 | 0.977 | 0.952 |
| Log_2_ VEGF R2 | 11.750 | 11.773 | -0.023 | 0.890 | 0.977 | 0.984 |
| Log_2_ FGF-4 | 10.875 | 10.850 | 0.026 | 0.912 | 0.977 | 1.018 |
| Log_2_ BMP-7 | 6.449 | 6.467 | -0.019 | 0.947 | 0.977 | 0.987 |
| Log_2_ b-NGF | 4.067 | 4.047 | 0.020 | 0.952 | 0.977 | 1.014 |
| Log_2_ HB-EGF | 4.725 | 4.722 | 0.004 | 0.991 | 0.991 | 1.003 |

IGFBP, insulin-like growth factor binding protein; MCSF R, macrophage colony-stimulating factor 1 receptor; SCFR, mast/stem cell growth factor receptor Kit; VEGF-D, vascular endothelial growth factor D; BMP-4, bone morphogenetic protein (BMP) 4; VEGF: vascular endothelial growth factor; EGF R, epidermal growth factor receptor; GDF-15, growth/differentiation factor 15; bFGF, basic fibroblast growth factor; NT-3, neurotrophin-3; OPG, osteoprotegerin; GH, growth hormone; SCF, stem cell factor; PDGF-AA, platelet-derived growth factor alpha polypeptide; IGF-1, insulin-like growth factor 1; BDNF, brain-derived neurotrophic factor; TGFa, transforming growth factor alpha; VEGF R3, vascular endothelial growth factor receptor 3; VEGF R3, VEGF receptor 3; PlGF, placenta growth factor; NGF R, nerve growth factor receptor; AR, amphiregulin; EG-VEGF, endocrine-gland-derived vascular endothelial growth factor; GDNF, glial cell line-derived neurotrophic factor; FGF-7, fibroblast growth factor 7; VEGF R2,vascular endothelial growth factor receptor 2; b-NGF, beta-nerve growth factor; HB-EGF, heparin-binding EGF-like growth factor. Adj.P.Val, BH method adjusted p-value; AveExp.VVS, the average plasma protein concentration of VVS group; AveExp.Control, the average plasma protein concentration of control group; Plasma protein concentration is expressed on a log2-scale.
